# Relationship between participation in leisure activities and constraints on Taiwanese breastfeeding mothers during leisure activities

**DOI:** 10.1186/1471-2458-13-410

**Published:** 2013-04-30

**Authors:** Hsueh-wen Chow, Yin-Han Dong

**Affiliations:** 1 Graduate Institute of Physical Education, Health & Leisure Studies, National Cheng Kung University, No1, University Road, Tainan, 701, Taiwan

## Abstract

**Background:**

Participation in leisure activities strongly associates with health and well-being. Little research has explored the relationship between participation in leisure activities and constraints on breastfeeding mothers during leisure activities. The purposes of this study are: 1) to investigate constraints on breastfeeding mothers during leisure activities and participation in leisure activities; 2) to investigate the differences between preferences for leisure activities and actual participation by breastfeeding mothers; 3) to segment breastfeeding mothers with similar patterns, using a cluster analysis based on the delineated participation in leisure activities and leisure preferences; 4) to explore any differences between clusters of breastfeeding mothers with respect to socio-demographic characteristics, breastfeeding behaviours and leisure constraints.

**Methods:**

This study has a cross-sectional design using an online survey conducted among mothers having breastfeeding experiences of more than four months. The questionnaire includes demographic variables, breastfeeding behaviours, preferences for leisure activities participation, and constraints on leisure activities. Collection of data occurred between March and July 2011, producing 415 valid responses for analysis.

**Results:**

For breastfeeding mothers, this study identifies constraints on breastfeeding related to leisure activities in addition to the three traditional factors for constraints in the model. This study demonstrates that reports of constraints related to children, family, and nursing environments are the most frequent. Breastfeeding mothers in Taiwan participate regularly in family activities or activities related to their children. Cluster analysis classified breastfeeding mothers into Action and Contemplation groups, and found that mothers within the latter group participate less in leisure activities and experienced more constraints related to breastfeeding.

**Conclusions:**

Implications provide a developmental design for public health policies for nursing-friendly environments to increase opportunities for breastfeeding mothers to engage in leisure activities and suggest various types of activities to increase participation of that population.

## Background

Considerable scientific research has proven the health benefits from breastfeeding accrue to both infants and mothers. For example, breastfeeding mothers have a lower rate of breast cancer [1,2], ovarian cancer [3-5], and osteoporosis [6]. For babies, breastfeeding has been proven to provide a protective mechanism against sudden infant death syndrome [7] and chronic diseases later in life [8]. Furthermore, these babies have lower rates of gastrointestinal and respiratory diseases [9,10]. In addition to the health benefits, the emotional, psychological, and societal advantages offered by breastfeeding cannot be underestimated [11,12].

The prevalence of breastfeeding is increasing internationally because of the evidence of the benefits, and the declarations of the Innocenti Declaration by the World Health Organization and United Nations Children’s Fund in 1990. However, the number of mothers choosing to breastfeed remains low in Taiwan. In a national study conducted in 2004, those that is breastfeeding, exclusively breastfeeding infants with only breast milk without other foods or liquids [13], in Taiwan was 33.21% when the baby was one month old, 16.93% for four–month old, and 13.13% at six-month old [14]. Although these statistics increased substantially to 58.8%, 36.2%, and 26.3%, respectively, in 2010 [15], mothers in Taiwan tend to cease breastfeeding earlier than the World Health Organization’s recommendation to continue exclusively breastfeeding for the first six months [16].

Although several studies in the nursing area [17] investigated the factors affecting the continuity of breastfeeding, including family and social support [18], employment status [19,20], types of employment [17], sleep deprivation [21,22], and fatigue [23,24], none of these studies explored the relationship between breastfeeding and women’s leisure.

Leisure is essential to well-being and health [25]. Several studies of leisure activities confirmed that leisure is a crucial factor that influences the health of women from several aspects. For example, regarding physical health, increased leisure activity can decrease the occurrence of coronary heart diseases and stroke [26], and for psychological health, participation in leisure activities can reduce stress through coping mechanisms, decreased feelings of anxiety, loneliness, and depression [27-29]. Leisure is also a vital context and channel for social support and social interactions [30,31]. In addition, since more than 20% of mothers have experienced symptoms of postpartum depression [32], leisure might be an important outlet, especially for mothers’ management of depression and enhancement of health.

Despite the health benefits of leisure activities for women, participation remains lower compared to male counterparts, according to several reports [33,34]. Several reasons contribute to this phenomenon, such as lack of opportunities for learning leisure skills [35] or low socioeconomic status [34]. Traditionally, researchers of leisure activities classified three factors impeding willingness or opportunities to participate in leisure activities: intra-personal, inter-personal, and structural constraints on leisure activities. Constraints on intrapersonal activities refer to individual psychological characteristics that impede willingness to participate in leisure activities. Inter-personal constraints for participating in leisure activities are those factors involving peers or groups, such as a lack of a partner. Structural constraints are issues such as inclement weather, cost, lack of opportunity, etc. [36]. Women’s leisure differs considerably from that of men’s leisure, and varies in diverse life situations. While several studies investigated leisure constraints and participation in leisure activities among mothers after giving birth or those with young children, examination of specific circumstances and situations, such as experiences during breastfeeding are necessary to understand, fully, the leisure experiences and constraints for women [37].

Several barriers may relate to participation of breastfeeding mothers during leisure activities; however, scant research has investigated the relationship between participation in leisure activities and constraints on breastfeeding mothers during leisure activities. Consequently, the objectives of this study are: a) to investigate the constraints on leisure activities and participation in leisure activities experienced by breastfeeding mothers; b) to investigate the differences between preferences for leisure activities and actual participation among breastfeeding mothers; c) to examine the differences among various demographic variables relating to participation in leisure activities, and d) to identify any patterns among breastfeeding mothers, using cluster analysis based on their participation in leisure activities and leisure preferences; e) to explore differences among clusters of breastfeeding mothers with respect to socio-demographics, breastfeeding behavioural variables and leisure constraints.

## Method

### Data collection

Several breastfeeding mothers indicated, in focus group discussions of previous research, confining limitation from nursing schedules and the inconvenience of leaving home. Consequently, they rely on the Internet to search for information or to communicate with others. Hence, the developed questionnaire uses an online survey application, provided by Google Docs. The survey appeared as a post on seven women- and baby-related websites popular with mothers in Taiwan. Data collection occurred between March and July 2011, resulting in 531 responses. Given that the 2010 census indicates 166,886 newborn babies in Taiwan, and a breastfeeding interval of four months, postpartum, was 53.6%, the targeted population for this study is 89451. The minimum sample size required with a 95% confidence level is 382. The analysis uses 415 responses, after eliminating invalid respondents and those not meeting the study’s criteria of breastfeeding babies for more than four months. The four months breastfeeding duration represents a set inclusion criterion because a national breastfeeding survey indicated that the breastfeeding rate decreases dramatically after four months [38]. This study obtained the approval from the Institutional Review Board (IRB) from National Cheng Kung University Hospital (ER-98-281).

### Research instrument

To conduct this study, an extensive literature review, and consultation with experts determined the factors that influence participation in, and constraints on, breastfeeding mothers during leisure activities. A pilot study in paper format contacted 14 local women to ensure ease of understanding and appropriateness of questions. The final questionnaire included four sections with variables for: socio-demographic data, preferences and participation in leisure activities, constraints on participation in leisure activities, and a short Taiwanese version of the International Physical Activity Questionnaire. This study analyzes data from the first three sections.

#### Socio-demographic and breastfeeding behaviour variables

A search of the literature indicated that age, family members, primary caregiver, educational level, employment status, number of children, age of children, breastfeeding methods, and breastfeeding duration may influence women’s willingness and duration to breastfeed [39-41] or women’s participation in leisure activities [33,42]. For the variable of exclusively breastfeeding, respondents responded to questions that indicate if their babies only receive breast milk, or a mix of breast milk and formula, during the duration of breastfeeding. The survey provided the four most popular practices for breastfeeding methods among Taiwanese mothers; respondents select the method that effectively described their situations. The practices are: 1) breastfeeding babies directly from their wet nurses; 2) expressing breast milk in advance and using bottles to feed babies; 3) alternating between feeding directly and bottle-feeding of expressed breast milk, and 4) feeding directly before bedtime, and bottle-feeding expressed milk at other times. Breastfeeding duration refers to the current length of time mothers continuously breastfeed. Since much of the behaviour or responses may change over time, the respondents were to indicate the most applicable situation at the moment of responding to the questionnaire.

#### Participation in leisure activities

This section is a modification of the study by Wu [43] of the typologies of women’s participation in leisure activities, which includes ten types: shopping (e.g. grocery shopping), children-related (e.g. activities associated or done with children), outdoor recreation (e.g. hiking, camping), intellectual activity (e.g. language learning, going to the library), indoor exercise (e.g. bowling, badminton), entertainment (e.g. going to pubs, watching movies), social activity (e.g. clubs, religious activities, banquets), art (e.g. painting, calligraphy), competition (e.g. Taekwondo), skill-related (e.g. swimming, golfing), and others. Respondents rated, on a five-point Likert scale, their preferences (from strongly dislike to strongly like) and actual participation frequencies (never, seldom, sometimes, often and always) for each type of leisure activity.

#### Leisure constraints

This section’s focus is constraints on leisure activities based on the conceptual model by Crawford & Godbey [36]. The model conceptualizes three types of constraints to participation in leisure activities which are addressed above. However, the current study adds ten constraints, influencing participation in leisure activities that relate to breastfeeding. Previous literature related to women [28,35,44] or breastfeeding [14,19,20], and previous qualitative research identify these impediments. In the questionnaire, 37 questions use a 5-point Likert-type scale. The possible responses ranged from 1 (*strongly disagree*) to 5 (*strongly agree*). Higher scores indicate that respondents perceive greater constraints. After factor analysis, four dimensions emerged and represent intrapersonal constraints, interpersonal constraints, structural constraints, and breastfeeding-related constraints, respectively. Each dimension has acceptable reliability with a Cronbach’s alpha range above 0.8 (see Table 1). The combined scale of Cronbach’s alpha was 0.931, and calculation of the score for each dimension of constraint is a summation of the score from each question and division by the number of questions.

**Table 1:** Factor analysis and t-test: reliability of constraints on leisure of breastfeed directly and two clusters of breastfeeding mothers

			**Breastfeeding practice**			**Clusters based on leisure preference and participation**		
**Statement constraints**	**Chronbach α**	**Total (N = 415)**	**Breastfeeding directly (N = 177)**	**All other practices (N = 238)**	**t-value**	**Action group (N = 231)**	**Contemplation group (N = 184)**	**t value**
**Intrapersonal constraints**	**.862**	**2.12**	**2.13**	**2.11**	**-.240**	**2.05**	**2.20**	−2.324*
Lack of interest		2.52	2.41	2.60	1.655	2.37	2.71	−3.019**
Lack of confidence		1.96	2.00	1.93	-.813	1.87	2.08	−2.437*
Afraid of going out alone		2.07	2.19	1.98	−1.936	2.04	2.10	-.503
Health concern		1.93	1.97	1.90	-.790	1.91	1.96	-.599
Difficult to get along with others		1.70	1.75	1.66	−1.256	1.65	1.75	−1.387
Unpleasant experience		1.97	1.99	1.94	.530	1.93	2.01	-.845
Not satisfied with self-body image		2.00	2.03	1.98	-.486	1.95	2.08	−1.320
Poor physical fitness		2.50	2.48	2.51	.258	2.44	2.57	−1.126
Lack of skill		2.41	2.36	2.44	.701	2.27	2.59	−2.952**
**Interpersonal constraints**	**.887**	**2.54**	**2.54**	**2.53**	**-.100**	**2.54**	**2.53**	**.106**
My family/friends oppose		2.15	2.14	2.15	.055	2.09	2.22	−1.196
No partner		2.75	2.76	2.73	.230	2.72	2.79	-.581
My partners are too busy at work		2.64	2.67	2.60	.631	2.66	2.62	.319
My partner are not available		2.26	2.26	2.26	-.075	2.31	2.19	1.295
My partners have difficulty traveling		2.31	2.38	2.26	−1.164	2.32	2.31	.128
My partners do not have time		3.12	3.14	3.09	-.424	3.10	3.14	-.343
My partners’ interests are different		2.69	2.68	2.69	.142	2.75	2.61	1.393
My partners do not have such leisure skills		2.39	2.39	2.38	-.040	2.38	2.40	-.187
**Structural constraints**	**.824**	**3.07**	**3.00**	**3.11**	**1.480**	**3.05**	**3.09**	**-.548**
Weather issues		3.23	3.28	3.20	-.786	3.34	3.10	2.144*
Taking care of family members		3.89	3.84	3.94	.955	3.91	3.87	.395
Too many chores		3.22	3.10	3.30	1.753	3.15	3.29	−1.198
Too much work		2.79	2.41	3.07	6.003***	2.72	2.89	−1.498
Lack of transportation		2.59	2.65	2.55	-.896	2.59	2.60	-.030
Lack of information		2.38	2.34	2.41	.711	2.32	2.45	−1.300
Lack of money		2.68	2.79	2.61	−1560	2.70	2.66	.392
Too crowded and too noisy		3.06	3.03	3.08	-.385	2.98	3.15	−1.463
Lack of time		3.63	3.43	3.78	3.105**	3.63	3.64	-.104
Lack of space/facilities		3.18	3.18	3.18	-.007	3.15	3.23	-.696
**Nursing-related constraints**	**.873**	**3.51**	**3.44**	**3.56**	**1.446**	**3.42**	**3.62**	−2.514*
Concern with babies’ health		3.42	3.38	3.44	.506	3.37	3.47	-.846
Babies too attached to mothers		3.76	3.91	3.65	−2.323*	3.75	3.78	-.265
No other family members could help with taking care of the babies		3.00	3.10	2.92	−1.345	2.92	3.09	−1.303
Leisure activities not appropriate for children		3.96	3.94	3.97	.383	3.95	3.97	-.138
Unfriendly nursing environment in leisure activities place/recreation space		3.87	3.73	3.98	2.232*	3.75	4.04	−2.676**
Feel guilty to bother other family members to take care of babies		3.80	3.67	3.89	1.970	3.75	3.86	−1.005
No or insufficient breastfeeding rooms in leisure activities place/recreation space		3.78	3.64	3.89	2.169*	3.64	3.95	−3.033**
Difficulty accessing breastfeeding rooms		3.78	3.61	3.90	2.489*	2.54	2.90	−2.797**
Embarrassment being outdoors and in others’ views		2.70	2.65	2.74	.701	2.88	3.14	−3.110**
Traditional myths from older generation		3.00	2.77	3.17	3.079**	2.86	3.07	−2.015*

Note: ^1.^ * p < .05, **p < .01, ***p < .001.

^2.^ Breastfeeding practice: Breastfeeding directly indicating breastfeeding their babies directly from wet nurse, other practice comprise feeding methods such as bottle feed with expressed milk, alternating between the breast directly and bottle with expressed milk, and directly from breast only before bedtime were combine together as all the others.

### Data analysis

Data, imported from the Google survey used SPSS version 17.0 for analysis. Descriptive statistics represent socio-demographic characteristics of respondents, with each variable investigated. Since the ages of breastfeeding mothers in this study represents a slight oversampling of the middle age group (30–34 years old), adopting weights to adjust for the selection probabilities for that age’s variable create a nationally representative sample. A bi-variate Pearson correlation and paired t-test for ten different types of leisure activities examined the correlations and differences between the preferences for leisure activities and actual participation. Conducting cluster analyses use the basis of preferences for leisure activities and actual participation of breastfeeding mothers to identify the existence of mothers’ patterns for comparison of a set of demographics with participation in leisure activities and profiles of leisure constraints. Cross-tabulation examined associations; Chi-squared statistics and t-test examined differences among clusters with regard to various demographic variables, breastfeeding behaviour variables, and constrains on participation in leisure activities. For variables violating Chi-Square test assumptions (the expected count in each cell is less than 5), Fisher Exact Test using open-source language R allowed further examination.

**Figure 1:**
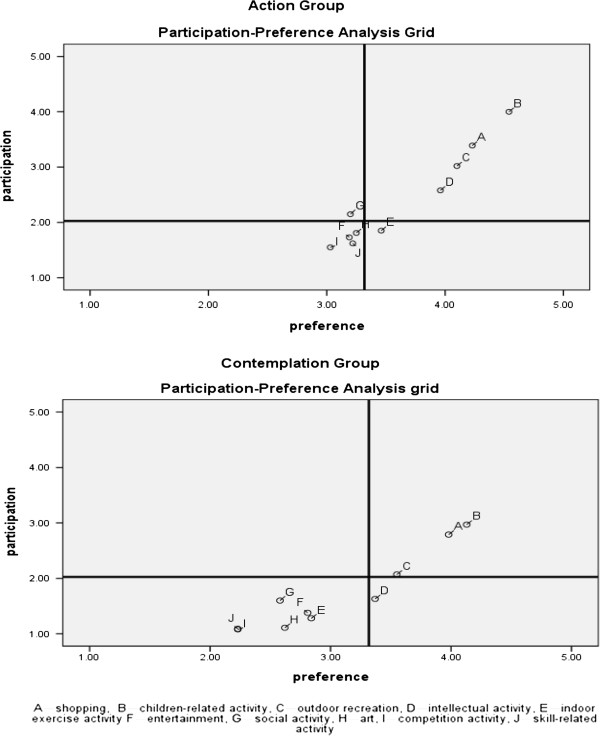
Leisure preference and leisure participation analysis grid of breastfeeding mothers in action group and contemplation group.

In addition, the study adopts the Importance-Performance Analysis technique [45] to compare different clusters of breastfeeding mothers’ participation in leisure activities and leisure preferences for participation in ten types of leisure activities. A graphic grid displays the calculation of participation in leisure activities and leisure preferences for each activity. Cross-hairs (vertical and horizontal lines), using the mean values of participation in leisure activities and leisure preference for all subjects, represent a calculation for separating derived factors into four quadrants (see Figure 1). The two-dimensional grid displays the level of participation in leisure activities on the vertical axis from, high (top) to low (bottom) and the magnitude of preference on the horizontal axis from, high (right) to low (left). Figure 1 illustrates the resulting representation of the data that produced the four quadrants (cells).

## Results

### Descriptive statistics of participants

The analyses use data from 415 valid respondents. The mean age of the respondents is 31.4 (±3.6). More than half of respondents live in the northern part of Taiwan. Approximately 14.9% of nursing mothers live with their parents, and 38.8% live with their parents-in-law. The children’s mothers are the primary caregivers (78.1%), and most of them have college degrees or higher education (88.7%), which is slightly higher than the national census data for the reproductive-aged population in Taiwan [46]. Of respondents, 58.1% have full-time employment; 63.4% have one child; 33.3% have two children, and 99.8% have a youngest child less than six years old. Regarding breastfeeding, 82.7% indicates breastfeed their babies only breast milk without formula, and for breastfeeding practices, 40.5% breastfeed directly; 16.6% breastfeed expressed breast milk from bottles; 31.8% adopt alternating breastfeeding, and 11.1% breastfeed directly just prior to bed time and use bottles for other times, such as during daily full-time work. The duration of breastfeeding varies; 40.2% breastfed for less than six months. A profile of detailed characteristics of respondents appears in Table 2.

**Table 2:** Characteristics of respondents

**Variable**	**Total (N = 415)**	
	**%**	**N**
**Ages**		
21-25yrs	6.0	25
26-30yrs	34.0	141
31-35yrs	45.3	188
36-40yrs	9.9	41
40+ and over	1.0	4
Unknown	3.9	16
**Education level**		
Junior high school	0.7	3
Senior high school	10.6	44
Vocational/college degree	68.9	286
Graduate school or above	19.8	82
**Job**		
Full-time	58.1	241
Part-time	3.9	16
Contracted	1.0	4
Unemployed	37.1	154
**Number of children**		
One	63.4	263
Two	33.3	138
Three	3.1	13
Four	0.2	1
**Age of youngest children**		
0-6yrs	99.8	414
7-12yrs	0.2	1
**Family member**		
Parents	14.9	62
Parents-in -law	38.8	161
Spouse	91.1	378
Children	91.6	380
Others	12.3	51
**Caregiver**		
Parents	17.3	72
Parents-in -law	22.4	93
Myself	78.1	324
Spouse	20.0	83
Babysitter	16.9	70
Others	2.4	10
**Exclusively breastfeeding**		
Mix breast milk and others	17.3	72
Only breast milk	82.7	343
**Breastfeeding practices**		
From breast directly	40.5	168
Bottle feed with expressed milk	16.6	69
Alternating between directly feeding and expressed milk from bottles	31.8	132
Directly from breast only before bedtime	11.1	46
**Breastfeeding duration**		
4-6 month	40.2	167
7-12 month	34.2	142
13-18 month	15.4	64
19-24 month	5.8	24
25-36 month	4.3	18

### Constraints on leisure activities

As shown in Table 1, in the total column (N = 415), breastfeeding mothers report that breastfeeding-related constraints on leisure are the most frequent encountered (Average Mean = 3.51), followed by structural constraints (Average M = 3.07), interpersonal constraints (Average M = 2.54), and intrapersonal constraints (Average M = 2.12). Items such as “Leisure activities are not appropriate for children (M = 3.96),” “Need to take care of family members (M = 3.89)” and “Unfriendly nursing environment during leisure activities’ place/recreation space (M = 3.87)” are the most frequently mentioned constraints indicated. Respondents also reported significant constraints from other breastfeeding related factors, such as “feeling guilty bothering other family members to care for the baby (M = 3.80),” “no or insufficient breastfeeding rooms in leisure activities’ place/recreation space (M = 3.78),” “difficulty accessing breastfeeding rooms (M = 3.78),” and “babies too attached to mothers (M = 3.76).” In order to examine is there any difference of leisure constraints in terms of their breastfeeding practice. Respondents were further divided into two groups which the first group refers to breastfeeding their babies directly from wet nurse, the second group comprises feeding methods such as bottle feed with expressed milk, alternating between the breast directly and bottle with expressed milk, and directly from breast only before bedtime were combine together as all the others. Significant differences were found in the following items: “Too much work”, “Lack of time”, “Babies too attached to mothers”, “Unfriendly nursing environment in leisure activities place/recreation space”, “No or insufficient breastfeeding rooms in leisure activities place/recreation space”, “Difficulty accessing breastfeeding rooms”, and “Traditional myths from older generation” (Table 1).

### Preferences for leisure and participation in leisure activities

The descriptive statistics with means and standard deviations of breastfeeding mothers’ leisure activity preferences and participation appear in Table 3, clarifying the differences between preferences for leisure activities or desire for leisure and actual participation in various types of leisure activities. The results reveal that most breastfeeding mothers in Taiwan participate in family activities or activities related to their children than other types of activities. The lowest preference and actual participation activities are skill-related and competitive. Although significant correlation coefficients in Table 3 reveal that participation is consistent with preferences (Pearson r ranges from 0.279 to 0.488), significant statistical differences appear between preferences and actual participation from paired t-tests for all ten types of leisure activities. The preferences for leisure activities of breastfeeding mothers are significantly higher than their actual participation.

**Table 3:** Mean differences in leisure participation and leisure preference in various leisure activities

**Leisure activities**	**Participation/preference**	**Mean (N = 415)**	**S. D.**	**t value**	**Pearson r**
Shopping	Leisure participation	3.07	.903	−23.083***	.365***
	Leisure preference	4.10	.683		
Children-related activity	Leisure participation	3.46	1.176	−17.236***	.488***
	Leisure preference	4.33	.665		
Outdoor recreation	Leisure participation	2.53	1.064	−23.209***	.279***
	Leisure preference	3.83	.799		
Intellectual activity	Leisure participation	2.07	.939	−32.177***	.323***
	Leisure Preference	3.67	.802		
Indoor exercise activity	Leisure participation	1.55	.751	−33.926***	.328***
	Leisure preference	3.18	.915		
Entertainment	Leisure participation	1.58	.750	−28.004***	.333***
	Leisure preference	3.02	1.015		
Social activity	Leisure participation	1.88	.976	−19.481***	.432***
	Leisure preference	2.87	.971		
Art	Leisure participation	1.44	.752	−33.252***	.362***
	Leisure preference	2.94	.866		
Competition activity	Leisure participation	1.31	.596	−31.018***	.383***
	Leisure preference	2.65	.917		
Skill-related activity	Leisure participation	1.35	.649	−29.369***	.373***
	Leisure preference	2.74	.933		
All	Leisure participation	2.02	.497	−47.455***	.338***
	Leisure preference	3.33	.485		

Note: ***p < .001.

### Classification of breastfeeding mothers

Classification of breastfeeding mothers based on preferences for leisure and participation in leisure activities employed cluster analysis, which identified two groups. The first group are those with a high level of preferences for leisure and participation in leisure activities. The second group of mothers represents those with high preference, but low levels of actual participation in leisure activities. Thus, the first group is named as the Action group, and the second group is named as the Contemplation group, since the first group participated in leisure activities, and the second group, despite high inclination displayed a low rate of participation in leisure activities.

### Differences between the action and contemplation group

Investigation of factors contributing to differences in the classifications of Contemplation and Action groups, used Crosstab Chi-square, and the results appear in Table 4. A significant difference exists between Action and Contemplation groups regarding their breastfeeding practices. Women in the Action group have a significantly higher percentage of instances for breastfeeding their babies directly than do those in the Contemplation group (χ2 = 9.004, df = 3, p = 0.029). No significant differences exist among age groups, education levels, working status, exclusively breastfeeding, and duration in comparison to leisure activity for the Contemplation and Action groups.

**Table 4:** Crosstabs of action group and contemplation group for various demographic and breastfeeding behavioural variables

**Variable**	**Action group**		**Contemplation group**	
	**%**	**N**	**%**	**N**
**Ages**	n = 226		n = 176	
21-25yrs	11.1	25	13.6	24
26-30yrs	36.7	83	33.0	58
31-35yrs	38.5	87	39.8	70
36-40yrs	12.8	29	12.5	22
40+ and over	0.9	2	1.1	2
	p-value = 0.8918			
**Education level**	n = 231		n = 184	
Junior high school	0	0	1.6	3
Senior high school	12.6	29	13.0	24
Vocational/college degree	71.0	164	64.1	118
Graduate school or above	16.5	38	21.2	39
	p-value = 0.1278			
**Job**	n = 130		n = 117	
Full-time	93.1	121	92.3	108
Part-time	6.2	8	6.0	7
Contracted	0.8	1	1.7	2
	p-value = 0.9173			
**Exclusively breastfeeding**	n = 231		n = 184	
Only breast milk	85.3	197	81.0	149
Mix breast milk and others	14.7	34	19.0	35
	χ^2^ = 1.368 d*f = 1*			
**Breastfeeding practice**	n = 232		n = 185	
From breast directly	48.7	113	34.6	64
Bottle feed with expressed milk	13.4	31	20.0	37
Alternating between the breast directly and bottle with expressed milk	28.4	66	34.0	63
Directly from breast only before bedtime	9.5	22	11.4	21
	χ^2^ = 9.004* d*f = 3*			
**Breastfeeding duration**	N = 231		N = 185	
4- less or equal to 6 month	39.8	92	44.9	83
7-12 month	33.8	78	31.9	59
13-18 month	15.2	35	14.6	27
19-24 month	7.3	17	4.3	8
25-36 month	3.9	9	4.3	8
	χ^2^ = 2.371 d*f = 4*			

Note: * p < .05.

### Constraints to participation in leisure activities among action and contemplation groups

Independent t-test investigated differences of constraints to participating in leisure activities between Action and Contemplation groups. Results reveal that the Contemplation group perceive significantly higher constraints from nursing-related (t = −2.514, *p* = 0.012) and intrapersonal leisure constraints (t = −2.324, *p* = 0.021). (See Table 1).

### Analysis of various leisure activities

As seen in Figure 1, ten types of leisure activities create plots for a preference-participation grid for Action and Contemplation groups. Both Action and Contemplation groups have similar perceptions toward children-related activities, shopping activities, and outdoor recreation, all represented in the high-preference and high-participation quadrant (top-right); entertainment, art, competitive activities, and skill-related activities represent the low-preference and low-participation quadrant (bottom-left) for both groups. However, intellectual activity, indoor exercise activities, and social activities have different perception for the Action group and the Contemplation group.

## Discussion

### Leisure constraints faced by breastfeeding mothers

The main purpose of this study is investigation of constraints breastfeeding mothers confront restricting participation in leisure activities. First, a factor structure of the constraints’ scale reveals that, in addition to the traditional three factors of constraints on leisure (intrapersonal, interpersonal, and structural constraints), a fourth factor, breastfeeding-related constraints, occur. This result presents a unique and specific constraint for breastfeeding mothers in daily life. Although several breastfeeding-related studies disclosed barriers influencing breastfeeding, none explored pursuing leisure. The uncovered factors result in limited and fragmented leisure time for breastfeeding mothers. The current study also demonstrates that constraints related to children, family, and nursing environments are, reportedly, the most intensively experienced.

Compared to men, women’s life stages and constraints, which may change over a lifespan considerably influence leisure activities [37]. In particular household obligations and family commitments, usually limit women’s time for leisure [47]. Mothers of young children tend to “put others’ wellbeing first” [48] instead of their own leisure needs. Although these family roles and obligations appear for consideration in many other studies of women and leisure [49,50], a lack of a sense of entitlement to leisure [51] is significantly intense for nursing mothers who feel responsible because their babies’ dependence on mothers for nourishment, particularly for breastfeeding mothers with children under six months old.

An unfriendly environment is another significant barrier for nursing mothers that inhibits participation in leisure activities. Several public and recreational areas do not have adequate nursing environments, which causes inconvenience for breastfeeding or to expressing milk. In 2008, Wu et al. interviewed Taiwanese nurses to gain insight into their breastfeeding experiences and found that outings are inconvenient most of the time, and embarrassment arises from breastfeeding in public; hence, the preference is to remain at home. If going outdoors with children, vehicles become mobile breastfeeding and milk-expressing “rooms” [17]. Breastfeeding in public has a privacy component due to limited nursing rooms available on-site at public leisure or recreational venues.

### Leisure preferences, participation in leisure activities, and comparison between action and contemplation groups

The ranking of leisure preferences for breastfeeding mothers are consistent with their participation in leisure activities. Most nursing mothers in Taiwan participate in more family activities or activities related to their children. The least preference and participation types are competitions and skills-related activities. The order suggests that women prioritize their time and activities to relate to children and families instead of individually oriented or skill-level activities. Further analysis from this study reveals that respondents’ preferences for leisure are considerably higher than their participation in all ten types of leisure activities. The difference between preferences or desire for leisure and actual participation re-confirms that constraints on leisure intervene.

From mapping of preferences for leisure and participation in leisure activities, for both Action and Contemplation groups, those activities strongly tied to family obligations, such as children-related activities, shopping (e.g. grocery shopping, baby –clothing shopping), and out-door recreation (e.g. taking children to a park) represent high-preference and high-participation activities. This result is consistent with many studies’ notions that women’s leisure typically involves work, caring for or, in the company of others, fulfilling roles as wives or mothers [52]. These activities may also represent a self-selected filter for activities because women choose leisure mainly based on a combination of opportunities available, as suggested by Colley [53].

A more in-depth analysis of the activities falling in the low-preference and low-participation quadrant (bottom-left), such as competitive or skill-related activities may also reflect the traditional concepts of gender that women avoid active forms of leisure or activities requiring skills. Conventional perceptions discourage women’s engaging in independent or active leisure and, perhaps lack opportunities to learn [52]. Altogether, the similar patterns illustrated for both top-right and bottom-left activities may relate to women’s ethic of care [51] in that, although they desire personal time to enjoy leisure, they feel guilty; according to Shaw, “Women may adjust their expectations to fit the reality of their lives” (p11) [54].

Interestingly, some activities appear in different quadrants. Intellectual activities, such as visiting museums or a library, and learning a language, have high degree of preference for both groups, but only the Action group demonstrates high participation. The Action group manages to attend these activities despite juggling time and obligations for feeding babies. Both groups report low participation in indoor exercise, but Action group perceives a higher preference. Explanation for this finding may arise from research suggesting breastfeeding can easily cause fatigue [55,56], but the Action group shows a desire to avoid lethargy. Actually, many women with young children do not meet the guidelines for physical activity to achieve health benefits [57]. Social activities, such as attending clubs, religious services, and participating in formal banquets, rate low in preference for both groups, but the Action group reveals a higher participation rate. Social activities listed in the questionnaire are more likely to be formal, which many breastfeeding women consider inappropriate if accompanied by children and breastfeed if necessary. The Action group might be those who are more out-going and participate in activities even if disagreeable.

### Comparison of demographic backgrounds, breastfeeding behaviours, and constraints on leisure: differences between action and contemplation groups

No significant differences appear in terms of breastfeeding mothers’ ages, educational levels, working status, and some breastfeeding behaviours. However, a higher percentage of women in the Action group adopted breastfeeding their babies directly, compared to other practices, such as bottle feeding with expressed milk, alternating between breastfeeding directly and bottle feeding with expressed milk, or directly feeding from the breast only before bedtime. The results also demonstrate that the Contemplation group has a significantly higher degree of constraint than the Action group for intrapersonal and breastfeeding-related leisure constraints. The explanation of these results could be that mothers, who adopted other breastfeeding practices other than directly breastfeeding, have more chores such as pumping breast milk, freezing and re-heating milk, washing bottles, etc. These tasks might take more time from mothers’ hectic lifestyles, which cause more stress for these groups of mothers and prevents them from participating in leisure activities and creates and unwillingness to leave home because not many nursing-friendly spaces exist in the public. Although different breastfeeding practices have not received significant attention in earlier breastfeeding-related research, recently, some articles began to debate the influence of different breastfeeding practices on women’s breastfeeding behaviour [58-60]. The influence of different breastfeeding practices on women’s perceptions of leisure warrant further investigation. Furthermore, many women lack interests or skills necessary for participation in leisure activities, which may be due to a lack of previous involvement in leisure activities. Many women perceive participation in leisure activities to be constrained because they have a narrow range of activities deemed appropriate for women or girls. The Contemplation group seems to suffer more severely in this regard. Breastfeeding-related constraints on leisure refer to those psychological states that are specific to breasting mothers, and environmental barriers for nursing. Since breastfeeding offers unique bonding between mothers and their babies, occasionally breastfeeding mothers may hesitate to separate from their babies because the mother is the only providers of nutrients or comfort. Mothers tend to abandon participation in leisure activities when experiencing internal conflict between personal fulfillment and obligation to the needs of others [61,62]. The Contemplation group might feel guilt/anxiety when engaging in personal leisure rather than in fulfilling obligations to others. They may lack a sense of entitlement for personal leisure [51]. Another crucial issue is nursing environment. Current literature related to nursing environments only focuses on work-sites [17,63-65]; limited attention accrues to providing adequate nursing environments for breastfeeding mothers in leisure/recreation settings, a crucial space for breastfeeding mothers’ participation in leisure activities, social participation, or time with children and family outside the home. Inferior facilities and services for breastfeeding mothers and inadequate promotion of breastfeeding in the public all relate to non-participation in leisure by breastfeeding mothers.

## Conclusion

This study indicates the constraints on leisure activities limit Taiwanese breastfeeding mothers’ access to participation in leisure activities, or preferences for actual participation in leisure activities. The study provides an awareness of constraints on leisure activities for breastfeeding mothers, and these issues have rare investigation. Several of these constraints on leisure are unlikely to be overcome by individuals alone.

The success of cluster analysis in identifying two groups of breastfeeding mothers with encountered constraints on leisure suggests that the approach may be useful in public health promotion using different strategies to enhance breastfeeding mothers’ leisure activities and participation. Breastfeeding is a challenge and requires support from family or health providers, and also society. The identification of specific constraints for breastfeeding mothers can provide evidence when vocalizing the effects of encountered constraints and empower them to negotiate rights for leisure. In addition, the data also offer insights for effective planning, provision, and management of leisure services and public health.

### Limitation of the study and directions for future studies

Several limitations require acknowledgement. First, the study only investigates constraints on leisure from mothers with breastfeeding experience. Since most other studies only focus on mothers with young children without specifying breastfeeding [47,56], this study could not yield differences between breastfeeding-mothers and non-breastfeeding mothers with regard to participation in leisure activities and constraints on leisure. Second, mothers might alter their participation in leisure activities or encounter different constraints during various stages of breastfeeding practice. The impact of breastfeeding on a mother’s participation in leisure activities and constraints on leisure might have different degrees of influence. In-depth qualitative research is necessary to explore these changes. Next, the study utilizes an online survey, which may induce a bias for those with higher educational status than the general reproductive population. Finally, the results of this study may be subject to cultural interpretations since it is exclusive to Taiwan. Since several leisure experiences and constraints relate to an individual’s social contexts [51], perhaps a noteworthy determination would be identifying any differences occurring in constraints on leisure confronted by nursing mothers in other settings. For example, a comparative study can interview mothers originally from Taiwan, but live in another country. Another study may examine the differences in constraint on leisure for breastfeeding mothers of various countries or ethnicities. Considerable knowledge regarding the unique situations that breastfeeding mothers face with regard to leisure activities is not available. By understanding these phenomena, offering a superior quality of life and improving the health of breastfeeding mothers and their families is possible.

## Competing interests

The authors declare that they have no competing interests.

## Authors’ contributions

HWC conceived the design of the study and drafted the manuscript. YHD collected the data and analyzed the data. Both authors have read and approved the final manuscript.

## Pre-publication history

The pre-publication history for this paper can be accessed here:

http://www.biomedcentral.com/1471-2458/13/410/prepub
